# G-fibre cell wall development in willow stems during tension wood induction

**DOI:** 10.1093/jxb/erv358

**Published:** 2015-07-28

**Authors:** Cristina Gritsch, Yongfang Wan, Rowan A. C. Mitchell, Peter R. Shewry, Steven J. Hanley, Angela Karp

**Affiliations:** Rothamsted Research, West Common, Harpenden, Hertfordshire AL5 2JQ, UK

**Keywords:** CCRC-M38, fasciclin, (1–4)-β-D-galactan, gelatinous fibres, homogalacturonan, immunofluorescence, immunogold, *in situ* hybridization, LM5, LM10, LM21, mannan, reaction wood, tension wood, TEM, *SxFLA12*, *SxCOBL4*, willow, xylan.

## Abstract

Willows (*Salix* spp.) are important as a potential feedstock for bioenergy and biofuels. Previous work suggested that reaction wood (RW) formation could be a desirable trait for biofuel production in willows as it is associated with increased glucose yields, but willow RW has not been characterized for cell wall components. Fasciclin-like arabinogalactan (FLA) proteins are highly up-regulated in RW of poplars and are considered to be involved in cell adhesion and cellulose biosynthesis. COBRA genes are involved in anisotropic cell expansion by modulating the orientation of cellulose microfibril deposition. This study determined the temporal and spatial deposition of non-cellulosic polysaccharides in cell walls of the tension wood (TW) component of willow RW and compared it with opposite wood (OW) and normal wood (NW) using specific antibodies and confocal laser scanning microscopy and transmission electron microscopy. In addition, the expression patterns of an FLA gene (*SxFLA12*) and a COBRA-like gene (*SxCOBL4*) were compared using RNA *in situ* hybridization. Deposition of the non-cellulosic polysaccharides (1–4)-β-D-galactan, mannan and de-esterified homogalacturonan was found to be highly associated with TW, often with the G-layer itself. Of particular interest was that the G-layer itself can be highly enriched in (1–4)-β-D-galactan, especially in G-fibres where the G-layer is still thickening, which contrasts with previous studies in poplar. Only xylan showed a similar distribution in TW, OW, and NW, being restricted to the secondary cell wall layers. *SxFLA12* and *SxCOBL4* transcripts were specifically expressed in developing TW, confirming their importance. A model of polysaccharides distribution in developing willow G-fibre cells is presented.

## Highlight

Immunolocalization of cell wall polysaccharides in gelatinous fibres of willow tension wood indicates a distinct distribution with a particular enrichment of (1–4)-β-D-galactan in the G-layer.

## Introduction

Willows (*Salix* spp.) are fast-growing shrubs and trees that have become the subject of much breeding and research due to interest in their cultivation as short-rotation coppice to provide sustainable biomass for the bioenergy and biofuel industries (Karp *et al.*, 2011). As reported for many trees, willows also react to mechanical and environmental stresses by modifying their growth to form reaction wood (RW) (Barnett *et al.*, 2014; Du and Yamamoto, 2007; Felten and Sundberg, 2013). In addition to being of interest to cell biologists as a striking example of dynamic cell wall modification, RW has industrial significance. The pulp and paper industry considers RW a nuisance because its presence reduces the quality of the wood products. For biofuel production, however, RW is advantageous, as it is associated with increased glucose yields following enzymatic saccharification (Brereton *et al.*, 2011, 2012). Of particular interest in willow is the novel finding that the ability to respond to conditions that induce RW varies among different genotypes, and that variation in this trait alone can account for the variation in the amount of glucose released (Brereton *et al.*, 2012). Further knowledge on the development and cell wall composition of RW in willows is therefore required to determine how specific genotypes may be used in specific environmental conditions in order to optimize the production of biofuels.

RW is a generalized term that is used to describe the specialized tissue that is formed naturally in trees in response to environmental conditions such as wind, or vertical displacement from growth on slopes and inclines, and can also be artificially induced by growing plants at an angle (Barnett *et al.*, 2014; Du and Yamamoto, 2007; Timell, 1986). RW can vary in composition but it typically comprises a specialized type of tissue that forms on the lower side of stems and branches of gymnosperms, called compression wood (CW), and on the upper side of stems and branches in angiosperms, where it is known as tension wood (TW) (Barnett *et al.* 2014). The opposite side of the stem/branch [opposite wood (OW)] is less well characterized but differs from the normal wood (NW) found in trees in which RW is absent. The modifications that occur in CW and TW in response to gravitropic, mechanical, and physiological stimuli provide the means for trees to adjust their growth and reorient their stems and branches. This response is associated with increased cambial activity, which in turn leads to asymmetric growth so that the TW or CW side of the stem is typically much wider than the OW side (Ruelle, 2014). In addition to eccentricity (asymmetric radial expansion of the stem), specific anatomical and cellular changes can occur, such as a decrease in vessel density and porosity and an increase in the fibre and xylem vessel length, as reported on the TW side of poplar stems (Jourez *et al.*, 2001). In artificially inclined willow stems, Brereton *et al.* (2015) have recently reported a marked decrease in vessel frequency, which was accompanied by a great increase in total vessel volume. However, there is considerable variability in the anatomical characteristics and the extent of CW and TW among plants and tissues due to specific growth stresses (Barnett *et al.*, 2014), as shown by studies on a large number of tropical species (Clair *et al.*, 2006; Ruelle *et al.*, 2006). Trees can also produce TW patches next to NW without induction, such as in the Raspalje poplar clone (Westing, 1965).

At the ultrastructural level, a typical feature associated with TW is the formation of a ‘gelatinous layer’ (G-layer) next to the lumen in xylary fibre cell walls; such fibres are called G-fibres. In NW without a G-layer, the fibre secondary cell wall (SCW) is characteristically composed of three layers (S1, S2, and S3). In TW fibres, the G-layer can partially or entirely replace the S3, sometimes the S2, and even the S1 layer of the SCW (Barnett *et al.*, 2014; Kwon, 2008; Nishikubo *et al.*, 2007; Wardrop and Dadswell, 1955). The G-layer is usually thick, rich in cellulose with cellulose microfibrils oriented almost parallel to the cell axis, and it is poorly lignified (Nishikubo *et al.*, 2007; Norberg and Meier, 1966; Pilate *et al.*, 2004), although in poplar lignin epitopes have been detected in substantial amounts in the G-layer (Joseleau *et al.*, 2004). Similar G-like fibres are found in a large number of angiosperms, such as flax, hemp, and ramie, and in many plant organs, whereas in trees their main role is in tensional stress to generate movement and provide structural support (Mellerowicz and Gorshkova, 2012). They can be present in bamboo culms (Liese, 1998; Liese and Grosser, 1971) and are produced in *Arabidopsis* by gravistimulation (Wyatt *et al.*, 2010). Because G-fibres have also been observed in a diverse group of non-dicot plants including coniferous species, they are thought to have appeared early in evolution and are more widespread than previously thought (Andersson-Gunneras *et al.*, 2006).

The particular chemical composition of G-fibres has been considered to be a desirable feature in plant material destined to be used as feedstock for saccharification due to its low lignification, low pentosan, and high cellulose contents (Mellerowicz and Gorshkova, 2012), which can lead to increased glucose yields (Brereton *et al.*, 2011, 2012). The polysaccharide composition of the cell wall matrix in the G-layers differs from that of primary and secondary cell walls (Fagerstedt *et al.*, 2014; Mellerowicz and Gorshkova, 2012). For instance, immunolocalization has shown that xylan, the major hemicellulose in SCWs, is absent in G-layers (Bowling and Vaughn, 2008). By contrast, the pectin rhamnogalacturonan I (RGI) containing side-chains of (1–4)-β-galactan (Mikshina *et al.*, 2013) has been considered to be a specific marker for CW of conifers (Altaner *et al.*, 2010) and TW. In poplar, (1–4)-β-galactan was exclusively detected in TW fibres and has been localized mainly in the interface between the SCW and the G-layer (Arend, 2008).

Homogalacturonan has also been detected in G-fibres in TW in a thin layer close to the lumen in sweetgum (*Liquidambar styraciflua*) (Bowling and Vaughn, 2008). Homogalacturonan is the most abundant pectic polysaccharide in plant cell walls, making up to 65% of pectin content; its main role is in cell adhesion. It is delivered to the cell wall in methylesterified form and then demethylesterified by pectin methylesterase, exposing acidic galacturonic residues. The extent of methylesterification is therefore important in changing the mechanical properties of the cell wall during growth (Wolf *et al.*, 2009). Immunocytochemical studies have shown that homogalacturonan is mainly found in the middle lamella and cell junctions of plant cells (Bowling and Vaughn, 2008; Christiaens *et al.*, 2011; Guillemin *et al.*, 2005; His *et al.*, 2001; Verhertbruggen *et al.*, 2009).

Mannans have structural and storage functions and could also play a role in RW formation. They comprise a group of abundant hemicelluloses, which are classified according to their backbone structure into two groups: (i) galactomannans and (ii) glucomannans and galactoglucomannans. While galactomannans are found in the cell walls of seed storage tissues (Ebringerová, 2005), galactoglucomannans are the main hemicellulose in softwoods, but are less abundant in angiosperm SCWs (Brett and Waldron, 1996; Ebringerová, 2005). In poplar, developing cells in NW at the primary cell wall stage may contain 1% glucomannan, whereas in mature NW the proportion increases to 5% (reviewed by Mellerowicz *et al.*, 2001). Although an overall decrease in glucomannan deposition has been reported in TW of poplar, the actual G-layer has been shown have a high glucomannan content (Kim and Daniel, 2012).

Among the most distinctive features of RW is the presence of fasciclin-like arabinogalactan (FLA) proteins containing a cell adhesion domain. *FLA*s are the most up-regulated transcripts in TW tissue of *Populus* as identified using microarray analysis (Andersson-Gunneras *et al.*, 2006). Fifteen poplar FLA proteins have been identified (Lafarguette *et al.*, 2004), 10 of which were especially and strongly expressed in mature xylem of TW, but not expressed in OW and cambial zone based on semi-quantitative PCR and abundance of expressed sequence tags. Analysis of FLA4 and FLA11/12 mutants in *Arabidopsis* suggested that FLAs have roles in cell expansion (Shi *et al.*, 2003) and stem biomechanics by affecting stem strength due to reduced cellulose synthesis in the cell wall (MacMillan *et al.*, 2010). However, their biological functions have not been clearly elucidated.

COBRA genes with two predicted cellulose binding sites are also highly differentially expressed in TW tissue of poplar (Andersson-Gunneras *et al.*, 2006), with 18 COBRA-like genes identified in the poplar genome (Zhang *et al.*, 2010). Their expression and functional analysis have been studied in more detail in *Arabidopsis*, rice, and maize. Analysis of mutants (*cob-4* and *cob-1*) in *Arabidopsi*s (Roudier *et al.*, 2005) suggested that COBRA protein, through its involvement in cellulose microfibril orientation, is an essential factor in highly anisotropic expansion during plant morphogenesis. COBRA-like proteins are also involved in stem strength in maize and rice (Ching *et al.*, 2006; Li *et al.*, 2003). However, the roles of FLA and COBRA in TW are not yet fully understood.

Although several immunocytochemical studies have investigated the composition of the cell wall in TW of poplar (e.g. Arend, 2008; Kim and Daniel, 2012), little has been published on the cell wall matrix polymer organization in willow TW. This study used confocal laser scanning microscopy and transmission electron microscopy (TEM) to determine the spatial distribution of a number of non-cellulosic cell wall polymers during TW induction in willow stems. In addition, in order to understand the molecular basis of FLA and COBRA genes in willow TW, the expression patterns of one FLA gene (*SxFLA12*) and one COBRA-like gene (*SxCOBL4*) were investigated using RNA *in situ* hybridization. The results provide a basis for future research and better understanding of how genotypes of willow may differ in their response to RW induction and their subsequent sugar release in biofuel production.

## Materials and methods

### Plant material

**Fig. 1.**
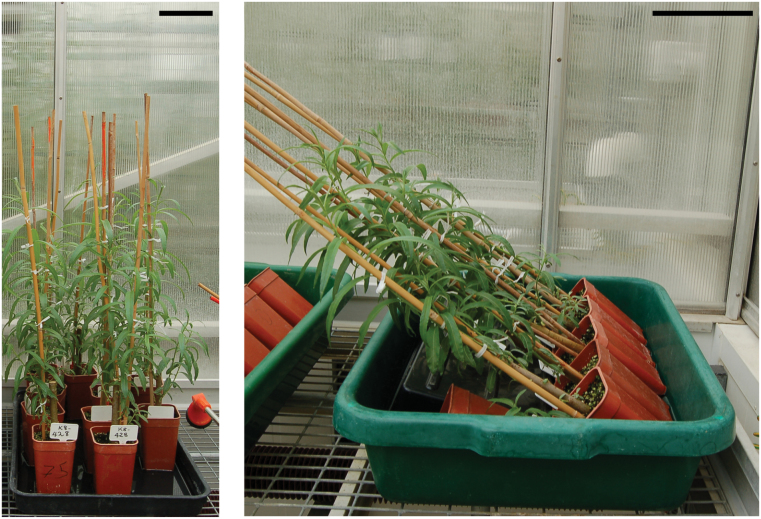
K8-428 willow plants. (A) Control upright plants. (B) Inclined plants for TW induction after 1 week of treatment. Bars: 10cm.

Plant material was grown under similar conditions to those described by Brereton *et al.* (2012). Stem cuttings measuring approximately 20cm in length × 10mm in diameter and containing an average of three axillary buds from K8-428 genotype <{*Salix viminalis* ‘Astrid’ × [*S. viminalis* ‘Astrid’ × (*S. schwerinii* × *S. viminalis* SW930984)] S3} × {*S. viminalis* ‘Astrid’ × [*S. viminalis* ‘Astrid’ × (*S. schwerinii* × *S. viminalis* SW930984)] R13}> were grown in Rothamsted standard compost mix in a glasshouse under a 16h day length regime. Two experiments were carried out. In Experiment I, the willow cuttings were grown for 4 weeks, after which TW was induced by inclining the stems to a 45° angle (Fig. 1). Samples were harvested from three replicate plants for fixation from the stem mid-point after 1 or 2 weeks of induction. As the diameter and hardness of the stems developed very quickly, in Experiment II, cuttings were grown for 2 weeks only prior to TW induction in order to facilitate their preparation for microscopy. Samples from this second experiment were collected after 4 weeks of induction. Control stems in both experiments were kept in an upright position. The growing tips were regularly tied to a supporting cane to maintain the correct orientation. *In situ* hybridization and immunolabelling results were found to be consistent between the two experiments.

### RNA extraction and probe labelling

RNA extraction was based on the method described by Chang *et al.* (1993). Frozen tissues were ground in liquid nitrogen and extracted in hexadecyltrimethylammonium bromide (CTA B) buffer [2% CTA B, 2% polyvinylpyrrolidone K30, 100mM Tris/HCl, pH 8.0, 25mM EDTA, 2.0M NaCl, 0.5g l^–1^ spermidine, 2% (w/v) 2-mercaptoethanol] with chloroform/isoamyl alcohol (24:1 v/v) to remove proteins. RNA was precipitated with 10M LiCl and incubated on ice overnight, dissolved in buffer [1.0M NaCl, 0.5% (w/v) sodium dodecyl sulphate, 10mM Tris/HCl pH 8.0, 1mM EDTA] to remove polysaccharides, and extracted once with chloroform/isoamyl alcohol (24:1 v/v). After ethanol precipitation, the total RNA was dissolved in diethylpyrocarbonate (DEPC)-treated water and stored at −80 °C.

*SxFLA12* and *SxCOBL4* were chosen to produce the *in situ* antisense and sense probes because they are the most highly expressed in TW based on microarray data (Andersson-Gunneras *et al.*, 2006) and the authors’ willow RNA-seq data (high-throughput sequencing of the entire complement of mRNA; unpublished). The sequences of target genes and alignment to poplar gene orthologues (*SxFLA12*, Potri.013G014200; *SxCOBL4*, Potri.004G117200) are shown in Supplementary Fig. S1 (available at *JXB* online). All probes were sequenced by Eurofins MWG Operon (London, UK) prior to RNA probe synthesis. The transcribed RNA was hydrolysed in carbonate buffer for 20–30min, precipitated in ethanol, and dissolved in DEPC water. Probes diluted 1:100 (2.6–3.6 μg ml^−1^) were used for hybridization. Probe efficiency was also tested by spotting the RNA probe on to positively charged nylon membrane with anti-digoxigenin (DIG) alkaline phosphatase-conjugated antibody and nitro blue tetrazolium chloride/5-bromo-4-chloro-3-indolyl phosphate, toluidine salt (NBT/BCIP) colour development.

### Sample preparation for microscopy

Sample preparation was carried out essentially as previously described by Wan *et al.* (2014). Stems were harvested from three control (upright) and three inclined plants. Cross-sections were cut with a razor blade from the middle of the stems at 1, 2, and 4 weeks after tipping and immediately fixed in 4% (w/v) paraformaldehyde in 0.1M Sorensen’s phosphate buffer (NaH_2_PO_4_.2H_2_O and Na_2_HPO_4_.12H_2_O buffer, pH 7) overnight at 4 °C. Samples were dehydrated in an ethanol series and embedded in either LR White hard grade resin (London Resin, TAAB) or paraffin wax (Paraplast Plus, Sigma). Transverse resin sections were cut with a diamond knife using a Reichert-Jung ultramicrotome for light microscopy, confocal microscopy, and TEM. Stem pieces of approximately 1.0cm in length were also fixed and sectioned with a Leica SM2010 sliding microtome at 80–100 µm thickness and stored in 70% ethanol until needed.

### Histochemical staining: identification of tension wood and lignification

G-fibres were identified by staining unembedded 100 µm cross-sections with 1% safranin O (in 50% ethanol) and 1% Chlorazol black E in methoxyethanol (Robards and Purvis, 1964), followed by dehydration in ethanol and permanent mounting in DPX mounting medium (Sigma 06522). Phloroglucinol-HCl stain was used to determine the degree of lignification in similar stem sections. Sections were imaged with a Leica MZ100 stereo microscope.

### Immunolabelling of cell wall epitopes

To determine the distribution of cell wall matrix epitopes [xylan, de-esterified homogalacturonan,(1–4)-β-D-galactan and mannans], 1 µm resin sections collected on Polysine slides were incubated in blocking solution [3 % (w/v) BSA (Sigma A7638) in PBS with 0.1% (w/v) Tween 20 (PBST)] for 1–2h. This was followed by 2–3h incubation in the primary antibodies (listed in Table 1) diluted 1:10 in 1% (w/v) BSA in PBST, either individually or in combination. After PBST washes, the sections were incubated for 1–2h at room temperature with anti-mouse or anti-rat AlexaFluor 488 or 633 secondary antibodies (Life Technologies) diluted 1:200 in 1% BSA in PBST. Some 100 µm unembedded sections were also treated similarly. Primary antibodies were omitted in control sections. After several washes with PBST, PBS and distilled H_2_O, some sections were stained with 0.01% Calcofluor White M2R (for cellulose staining) and mounted either in distilled H_2_O or anti-fade CitiFluor AF1 and examined with a Zeiss 780LSM confocal microscope. Brightness was adjusted using Photoshop CS5.1 to aid visualization of printed images.

**Table 1.** Primary antibodies used in this study.

Antibody	Target epitope	Source/references
LM5 (Rat IgG)	Linear tetrasaccharide in (1–4)-β-D-galactans	Plant Probes (Jones *et al.*, 1997)
LM10 (Rat IgG2)	Unsubstituted and relatively low-substituted xylans	Plant Probes (McCartney *et al.*, 2005)
LM21 (Rat IgM)	Binds to β-(1–4)-manno-oligosaccharides from DP2 to DP5. Recognizes mannan, glucomannan, and galactomannan polysaccharides	Plant Probes (Marcus *et al.*, 2010)
CCRC-M38 (Mouse IgG1)	De-esterified homogalacturonan	University of Georgia, Complex Carbohydrate Research Center (Pattathil *et al.*, 2010)

Transverse ultrathin sections at 100nm were also prepared for TEM from TW, OW, and NW of 4-week-old stems and collected on Formvar coated gold grids. The sections were treated with 3% BSA in PBS for 1h, incubated with the same primary antibodies diluted 1:5 in 1% BSA in PBS for 2h, washed three times with 1% BSA, followed by incubation in the secondary antibodies (goat anti-rat or anti-mouse IgG 10nm gold conjugates, AbCam) diluted 1:40 in 1% BSA in PBS for 1h, before final washes with 1% BSA in PBS and distilled H_2_O. Sections were post-stained with 2% (aq) uranyl acetate and examined with a Jeol JEM2010 transmission electron microscope.

### *In situ* hybridization

Transverse sections of 12–14 µm thickness were cut from wax-embedded samples with a Reichert rotary microtome, floated on DEPC-treated H_2_O at 40 °C, collected on to Polysine slides, and dried at 37 °C. Sections were dewaxed in HistoClear II, rehydrated in an ethanol series, digested with Proteinase K for 30 minutes at 37 °C, and post-fixed in 4% (w/v) paraformaldehyde in PBS (pH 7.4) for 10 minutes. Sections were acetylated for 10 minutes in 0.1M triethanolamine buffer with 0.5% (v/v) acetic anhydride, dehydrated in an ethanol series, and hybridized with the antisense and sense DIG-labelled RNA probes diluted 1:100 at 60 °C overnight. After several washes in 1 × SSC (saline sodium citrate buffer) and 50% formamide at 65 °C, the sections were treated with RNase A in NTE buffer (2mM EDTA, 0.5M NaCl, 1mM Tris/HCl) at 37 °C for 30 minutes, and then were incubated in 1% (w/v) blocking reagent (Roche) for 1h followed by incubation with anti-DIG alkaline phosphatase antibody conjugate (Roche) diluted 1:1600. The signal was detected with NBT/BCIP (Roche). A Zeiss Axiophot light microscope equipped with a Retiga Exi CCD digital camera (Qimaging, Surrey, BC, Canada) and MetaMorph software version 7.5.5 9 Molecular devices, Sunnyvale, CA, USA) were used to acquire the images under bright-field optics.

## Results

### Histology: tension wood identification and lignification

**Fig. 2.**
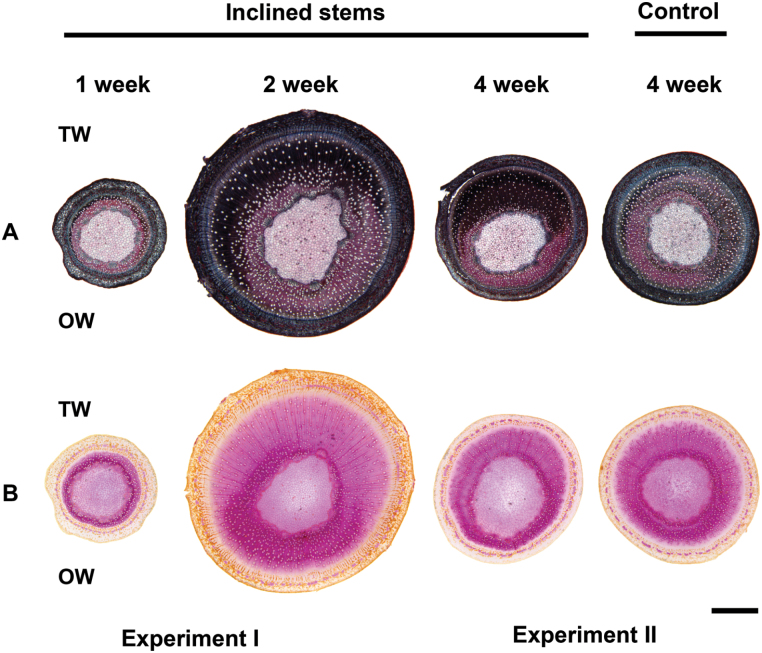
Histological staining. The stems inclined for 1 and 2 weeks were from Experiment I (cuttings grown for 4 weeks before induction) and stems inclined for 4 weeks were from Experiment II (cuttings grown for 2 weeks before induction). Because of the difference in growth rates between the experiments, the 2-week inclined stems are larger in diameter. (A) Chlorazol black and safranin stain of stem cross-sections. Black staining indicates the presence of G-fibres on the TW side. (B) Phloroglucinol-HCl stain. G-fibres on the TW side show reduced pink staining. Bar: 1000 µm.

Cuttings were grown for either 4 weeks (Experiment I) or 2 weeks (Experiment II) before induction, with cross-sections of 1- and 2-week induced plants being taken from Experiment I, and of 4-week induced plants and control samples from Experiment II. Because the plants in Experiment I were older before induction, the stems sampled after 2 weeks’ induction in Experiment I had larger diameters than those after 4 weeks in Experiment II. Staining with Chlorazol black and safranin showed that TW started to form from the first week after inclined growth (Fig. 2A). The G-layers were stained black with Chlorazol black (Fig. 2A), whereas in OW normal fibre cell walls were stained red with safranin. Following a longer period of induction in Experiment II, the area of TW increased greatly and G-fibres stained strongly, indicating high cellulose and low lignin content of the G-fibres. By contrast, NW in control stems were significantly less stained with Chlorazol black. Phloroglucinol-HCl stain showed that lignification was greatly reduced on the TW side (Fig. 2B) in contrast to OW, which was strongly stained pink due to higher levels of lignification.

### Localization of cell wall epitopes

Distinct patterns of distribution of cell wall pectic and hemicellulosic epitopes were observed during TW wood formation after 1, 2, and 4 weeks of TW induction. Patterns were similar for all stages but much more marked after 2 and 4 weeks of induction; therefore, in most cases only images of 4-week induced stems are shown. Immunolabelling results for tissues from OW and control straight stems were similar; therefore, mostly images of OW are shown. Negative control sections probed solely with secondary antibodies showed absence of labelling (Fig. 7O for TEM and Supplementary Fig. S2 for confocal immunofluorescence). Control sections without the primary antibodies showed no significant autofluoresence from lignin using the same confocal gain settings as for experimental sections (see also Supplementary Fig. S2).

### Double labelling with LM5 antibody [(1–4)-β-D-galactan)] and CCRC-M38 antibody (homogalacturonan)

**Fig. 3.**
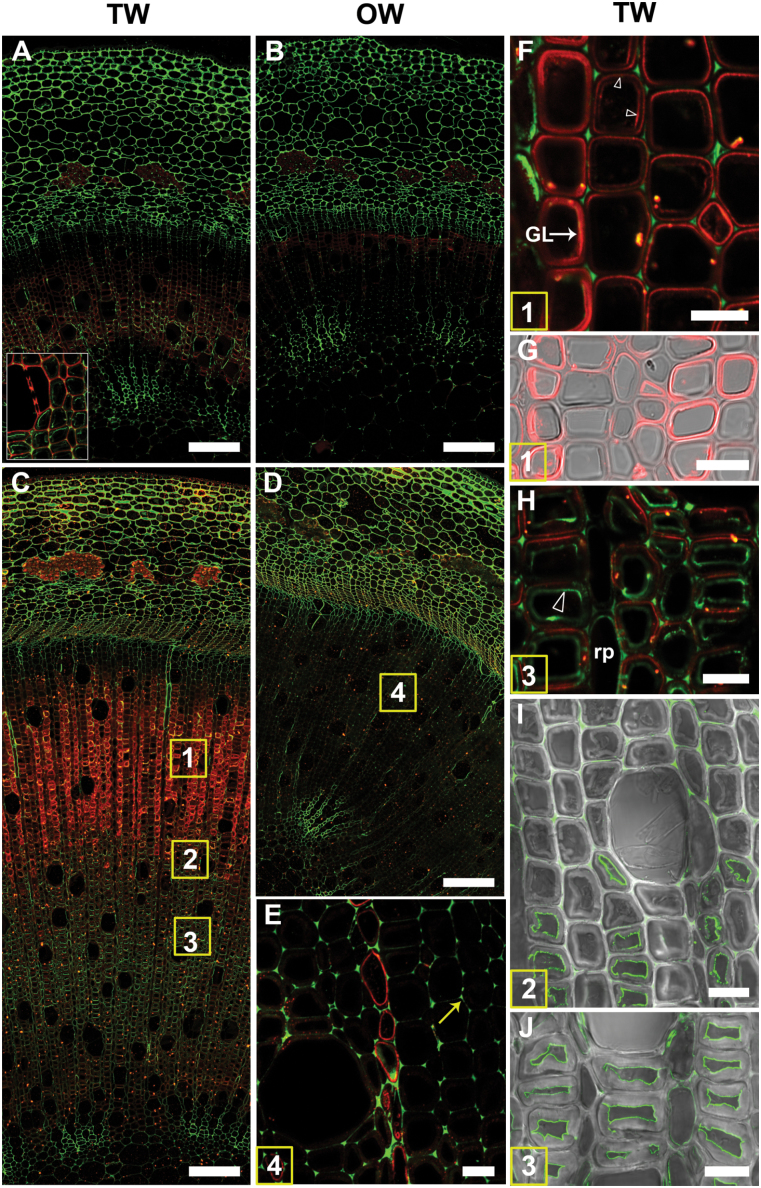
Localization of (1–4)-β-D-galactan (LM5 antibody in red) and homogalacturonan (CCRC-M38 antibody in green) in transverse sections of stems after 1 or 4 weeks of TW induction. Numbers in the squares in (C) and (D) indicate similar areas shown in more detail in subsequent images. (A) to (H) are images of 1 µm resin sections and (I) and (J) are images of 100 µm unembedded sections. (A) Stem after 1 week of tipping showing the distribution of (1–4)-β-D-galactan (red) in TW fibres and of homogalacturonan (green) in the more mature inner side of the stem (detail in the inset). (B) OW side of the same section showing virtually no fluorescence in xylary fibres with the LM5 and CCRC-M38 antibodies. (C) TW side of a 4-week tipped stem showing strong labelling of G-fibres with the LM5 antibody in the outer developing and maturing part of the stem. (D) OW side of the same stem in (C). Fibre cell walls are not labelled or only weakly labelled with the LM5 and CCRC-M38 antibodies. (E) Detail of area 4 in (D). There is no labelling of OW fibre cell walls, indicating the absence of (1–4)-β-D-galactan. Some labelling is seen in ray parenchyma. Homogalacturonan is detected in cell corners (arrow). (F) and (G) Detail of area 1 in (C). LM5 antibody shows differential distribution of (1–4)-β-D-galactan from area 1 in files of G-fibres. The G-layer is labelled in some fibres, whereas in others the interface between the G-layer and SCW is labelled (arrowheads). (H) Detail of area 2 in (C). LM5 is bound mainly to the SCW and CCRC-M38 labels mainly the inner lamella of the G-layer (arrowhead). (I) and (J) Similar areas to 2 and 3 in (C) of unembedded sections showing CCRC-M38 mainly bound to the inner lamella of the G-layer. GL, G-layer; v, xylem vessel; rp, ray parenchyma. Bars: A, B, C, D = 100 µm; E, F, G, H, I, J = 10 µm.

The overall distribution pattern of (1–4)-β-D-galactan revealed using the LM5 antibody was similar after 1 (Fig. 3A), 2, and 4 weeks of induction, but more distinct in older stems (Fig. 3C). Strongest binding of the LM5 antibody occurred in G-fibres of the developing and maturing xylem of TW in the outer half of the stem (Fig. 3C, red fluorescence). Close examination revealed labelling in the G-layers but binding was not homogeneous. Some G-fibre cell files were interspersed with rows of other fibre cells where LM5 labelling was confined to the SCW (Fig. 3F, G). There was no evidence that this was confined to any particular fibre area, such as tips. In the inner older mature G-fibres, (1–4)-β-D-galactan was detected only in the SCW and was generally absent from the G-layer (Fig. 3H). TEM observations were largely in agreement with immunofluorescence results, but more detail on the location of the β-galactan epitope was obtained. In general only one layer (S1) of SCW could be detected in G-fibres and two layers (S1 + S2) in normal fibres. In the early stages of G-layer deposition, some fibres exhibited abundant gold labelling in the G-layer itself, whereas in others the labelling was restricted to the interface between the SCW and the newly formed G-layer (Fig. 7 A, B, C). Fig. 7B shows binding of the LM5 antibody in a thin layer on the inner side of the lumen side of the SCW, where initiation of G-layer deposition is possibly occurring. In more mature G-fibres, a similar pattern was noted in which the G-layers of some fibres were enriched with (1–4)-β-D-galactan (Fig. 7 D, E); however, in the fully mature fibres the labelling tended to be more sparse in the G-layer or absent and generally restricted to the SCW/G-layer interface (Fig. 7 F). In contrast to TW, the fibre cell walls in OW and NW in control stems were either not labelled or poorly labelled as shown by immunofluorescence (Fig. 3 B, D, E). TEM also showed almost negligible levels of labelling of fibre SCWs in NW and OW (Fig. 7G).

Immunofluorescence revealed that the CCRC-M38 antibody (which recognizes de-esterified homogalacturonan) bound strongly to the cell corners of all cells in TW, OW and NW, and also to the compound middle lamella in the cells of the cortex (Fig. 3A–I, green fluorescence). As with (1–4)-β-D-galactan, the homogalacturonan epitope also exhibited a distinct distribution pattern between the outer maturing xylem and the inner mature xylem of TW (Fig. 3A, C). In the outer maturing G-fibres, labelling was mainly observed in the cell corners (Fig. 3 F), whereas in the mature fibres in the inner part of the stem it was also bound to the innermost cell wall lamella of the G-layer close to the lumen (Fig. 3H, I, J). TEM using immunogold confirmed that the homogalacturonan epitope was present in the cell corners and, to some extent, in the middle lamella of both TW (Fig. 7H, I) and NW (Fig. 7K) fibres. In old mature fibres with very thick G-layers, CCRC-M38 labelling was also often detected in the area of the G-layer next to the lumen (Fig. 7J), as also seen in Fig. 3 (J).

### LM10 (xylan)

**Fig. 4.**
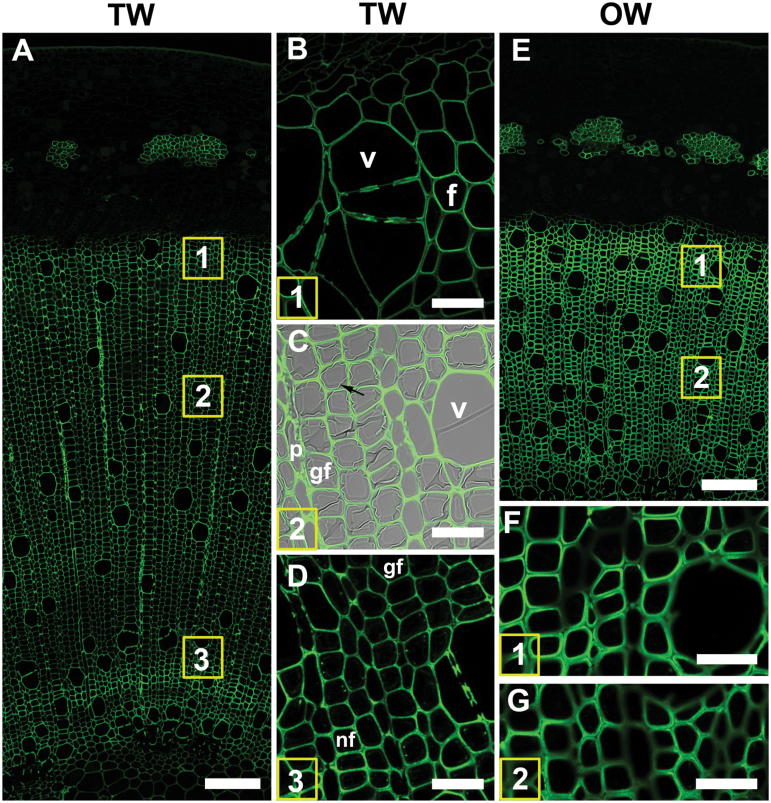
Xylan (LM10 antibody) distribution in TW and OW after 4 weeks of tipping. (A) TW side. Numbers indicate the areas shown in detail in B, C and D. (B) Detail of area 1. Differentiating xylem. (C) Area 2. Maturing xylem. The G-layer is not labelled (arrow). Antibody labeling is only present in secondary cell walls of G-fibres, vessels and ray parenchyma. (D) Area 3. Mature xylem. Normal fibres have thicker secondary cell walls than G-fibres. (E) OW of same section shows strong labelling of all fibre SCWs. (F) and (G) show detail of areas 1 and 2 in (E). All fibre SCWs are strongly labelled. (v) xylem vessel, (f) fibre, (rp) ray parenchyma. Bars: A, E = 100 µm; B, C, D, F, G = 20 µm.

Xylan was detected with the LM10 antibody in all SCWs in developing and mature xylem in TW, OW, and NW. The G-layer itself was not labelled, resulting in weaker fluorescence on the TW side (Fig. 4A, B, C). In OW, fluorescence was much stronger due to thicker SCWs (Fig. 4E, F, G). TEM confirmed these observations and showed labelling to be clearly confined to the SCWs (Fig. 7L, M, N).

### LM21 (mannans)

**Fig. 5.**
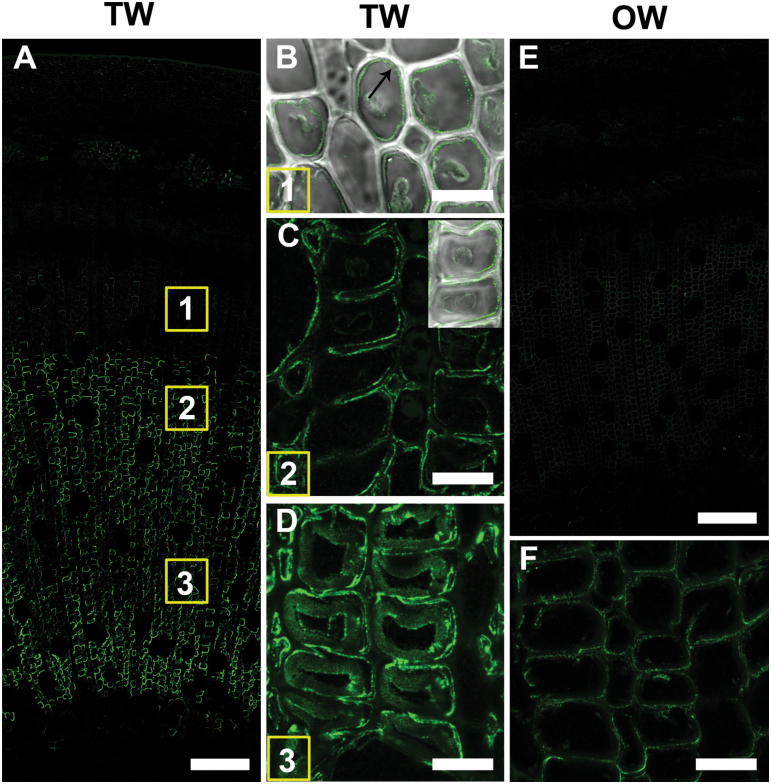
Mannan (LM21 antibody) distribution in TW and OW after 4 weeks of inclined growth. (A) 1 µm resin section of TW side showing strong binding of LM21 in the maturing and mature G-fibres. The numbers in the squares indicate similar areas to those shown in detail in B–D. (B) 100 µm unembedded section of developing G-fibres of area 1 showing a thin layer of labelling in the SCW/G-layer (arrow). (C) 100 µm unembedded section of more mature fibres showing labelling at the SCW/G-layer interface. (D) 100 µm unembedded section of very thick-walled mature G-fibres showing binding of the LM21 antibody in the SCW and also some more diffuse labelling in the G-layer. (E) 1 µm resin section of OW side showing very weak binding to fibre cell walls. (F) Detail of fibres in (E) showing light labelling of cell walls, possibly S1 layer of the SCW. Bars: A, E, 100 µm; B, C, D, F, 10 µm.

Immunofluorescence showed that the LM21 antibody was mainly bound to G-fibre cell walls of TW (Fig. 5 A), with labelling in the interface between the SCW and the G-layer in the developing and maturing fibres (Fig. 5B, C). In fully mature cells the G-layer itself was also labelled; however, the signal was weaker (Fig. 5D). At low magnification, no significant labelling could be seen (Fig. 5E) in OW, and at higher magnification only weak labelling was detected in the region that may be interpreted as being the S1 layer of the SCWs (Fig. 5F).

### Calcofluor white staining for cellulose

**Fig. 6.**
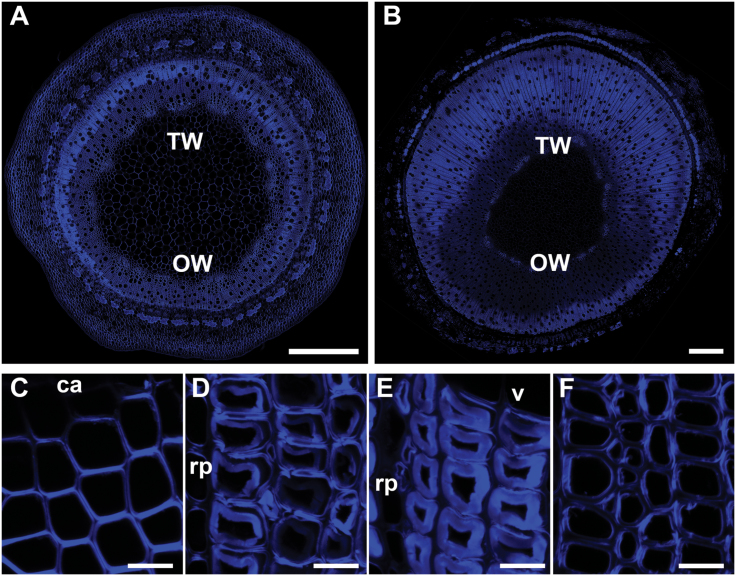
Transverse 100 µm thick unembedded stem sections stained with Calcofluor white showing the distribution of cellulose. (A) Stem after 1 week of TW induction. (B) Stem after 2 weeks of TW induction. (C) TW developing G-fibres after 4 weeks of inclined growth. (D) TW maturing G-fibres after 4 weeks of inclined growth. (E) TW mature G-fibres after 4 weeks of inclined growth. (F) OW maturing normal fibres after 4 weeks of inclined growth. ca, cambium; rp, ray parenchyma; v, xylem vessel. Bars: A, B = 500 µm; B, C, D, F = 10 µm.

Strong fluorescence was observed with Calcofluor white in the G-layers of TW after the first week of inclined growth (Fig. 6A). Fluorescence extended into the G-layers in conjunction with G-fibre maturation (Fig. 6B–E). In OW, fibres were not clearly stained because of the lack of the very thick G-layer (Fig. 6F).

**Fig. 7.**
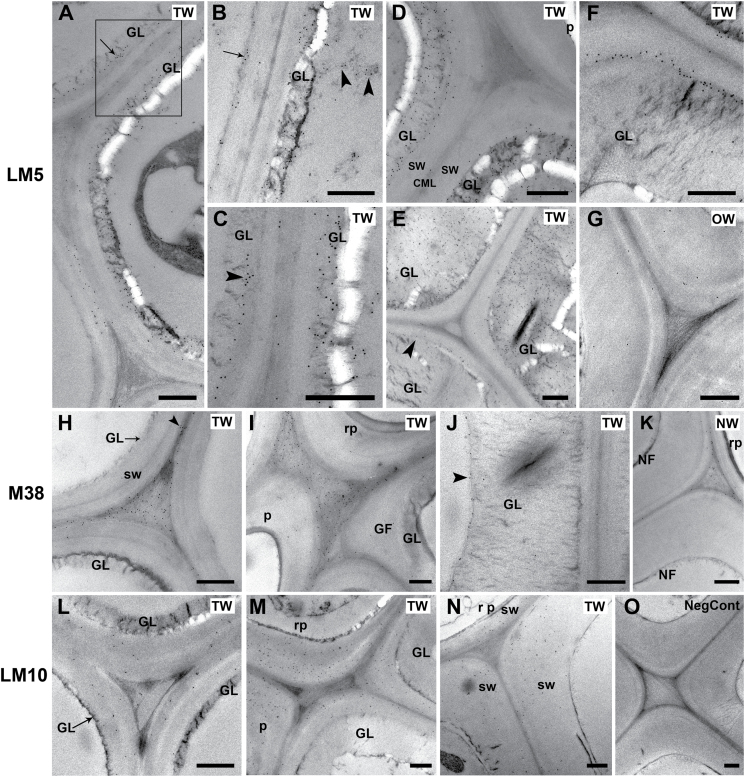
TEM immunogold labelling. (A–G) Localization of (1–4)-β-D-galactan with the LM5 antibody in TW (A–F) and OW (G) after 4 weeks of inclined growth. (A) Early G-layer formation. Binding occurs in the G-layer itself or in the interface between the SCW and the G-layer (arrow). The area in the square is magnified in (C). (B) Detail of two adjacent developing G-fibres at early development. In the G-fibre on the left, gold labelling shows that early deposition of (1–4)-β-D-galactan occurs on the inner side of the SCW before the G-layer itself is formed (arrow). The G-layer is already being formed on the adjacent cell and it is labelled. In addition, electron-dense areas in the lumen are also labelled (arrowheads). (C) Detail of the two adjacent fibres in the square in (A) showing differential LM5 labelling occurring in the interface between SCW and the G-layer (arrowhead) or in the G-layer itself. (D) Maturing G-fibres showing thicker G-layers strongly labelled with LM5 antibody next to a ray parenchyma cell with unlabelled SCW. (E) Mature G-fibres showing differential labelling with LM5. Labelling in one of the fibres is mainly in the interface between the SCW and the G-layer (arrowhead). The other two fibres exhibit strong labelling of the G-layer. (F) Fully mature G-fibre. Labelling in these fibres is mainly confined to the SCW-GL interface. (G) OW mature fibres. Very sparse gold labelling is observed in the SCWs of xylary fibres. (H–K) Localization of de-esterified homogalacturonan with the CCRC-M38 antibody in TW after 4 weeks of inclined growth (H–J) and NW in a vertically grown stem (K). (H) Developing G-fibres. Homogalacturonan is localized by the CCRC-M38 antibody in the compound middle lamella (arrowhead) and in the intercellular spaces, which are filled with fibrous material. (I) Homogalacturonan labelling in the intercellular spaces between parenchyma ray cells and G-fibres. (J) Mature G-fibre from the inner part of the stem where CCRC-M38 labelling is mainly confined to the inner lamella of the G-layer (arrowhead). (K) NW fibres walls in a straight stem. CCRC-M38 antibody labelling is present only in cell corners and compound middle lamella. (L–O) Localization of xylan with the LM10 antibody in TW (L–N) after 4 weeks of inclined growth and OW (O). (L) Developing G-fibres. Xylan is restricted to SCWs, but labelling is not very strong. (M) LM10 is bound to the SCWs of ray parenchyma and mature G-fibres. No labelling is detected in the G-layer. (N) OW. Labelling is also confined to the SCWs of fibres and parenchyma cells. (O) Negative control section probed with only the secondary 10nm gold anti-rat antibody. Straight stem fibres showing no gold labelling in any of the cell walls. GF, G-fibre; GL, G-layer; sw, secondary cell wall; rp, ray parenchyma; TW, tension wood; OW, opposite wood; NW, normal wood. Bars: 0.5 µm

### *In situ* localization of *SxFLA12* and *SxCOBL4* transcripts

**Fig. 8.**
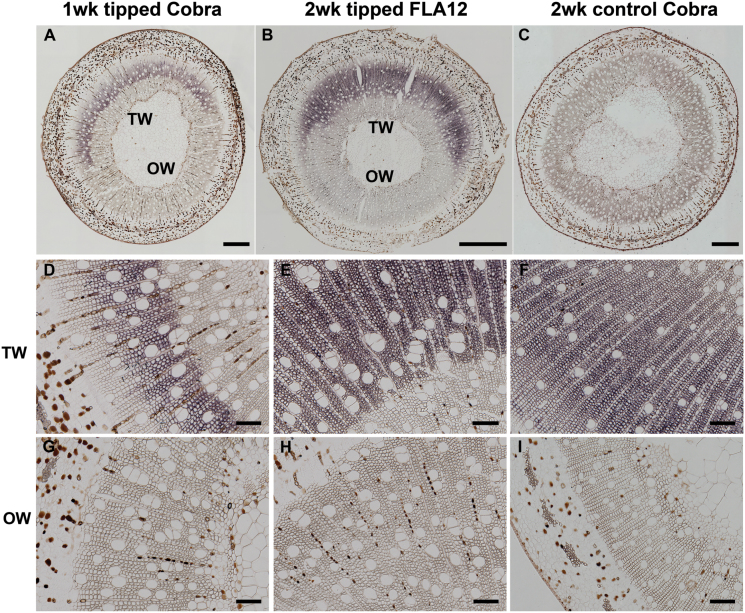
Expression of SxCobL4and SxFLA12 genes in transverse sections probed with antisense probes. (A) COBRA gene expression after 1 week of tipping is shown by the purple staining on the TW side of the stem. (B) A 2-week tipped stem showing that SxFLA12 gene expression occurs on the TW side of the stem. (C) Lack of purple colour in a control upright stem indicates no expression of the COBRA gene after 1 weeks of growth. (D) 1-week inclined stem shows strong expression of the COBRA gene in the G-fibres of the TW side. (E, F) After 2 and 4 weeks of tipping, respectively, strong expression of COBRA gene is shown in the G-fibres. (G–I) No COBRA gene expression is detected in OW of the same stems in D, E, and F, respectively. Bars: A–C: 100 µm, D–I: 100 µm.

Both *SxCOBL4* and *SxFLA12* transcripts were strongly expressed in G-fibres of TW (Fig. 8A, B, Supplementary Fig. S3) but absent in NW of straight control stems (Fig. 8C). Expression started after 1 week of inclined growth and increased after 2 and 4 weeks of inclined growth in newly formed G-fibres as well as in mature fibres (Fig. 8). By contrast, no signal was detected in OW (Figure 8A, B, G, H, I) and in NW of control stems (Fig. 8C). Light staining was occasionally observed in control stems, probably due to the development of some G-fibres resulting from natural movement of the stem (Supplementary Fig. S3). No hybridization was detected using the sense probes in both NW and TW (Supplementary Fig. S3) and control straight stems.

## Discussion

Four monoclonal antibodies were used to analyse the distribution of pectic [homogalacturonan and (1–4)-β-D-galactan] and hemicellulosic (xylan and mannan) epitopes in TW of willow stems induced for up to 4 weeks. The expression of *SxFLA12* and *SxCOBL4* transcripts during TW formation were also determined for the same samples.

Immunolabelling showed that (1–4)-β-D-galactan was primarily present in the G-layer of fibres located in the developing and maturing xylem in the outer part of the stem. Labelling of the G-layer was not homogeneous and was found to alternate between files of G-fibres. TEM confirmed this finding and also showed labelling of the interface between the SCW and the G-layer. By contrast, labelling in OW or NW was virtually absent from fibre SCWs. These observations suggest that (1–4)-β-D-galactan performs a specific function in TW of willow.

Work on poplar TW has demonstrated that both the area of fibres with G-layers per unit of tissue area and the thickness of the G-layers are influenced by the degree of the growth stress (Fang *et al.*, 2008; Yamamoto *et al.*, 2005). Therefore, it is clear that the G-layer plays a major role in producing tensile stress and, although there is no agreement regarding the underlying mechanism, several hypotheses have been put forward (Fagerstedt *et al.*, 2014; Mellerowicz and Gorshkova, 2012). One hypothesis suggests that radial swelling of the G-layer puts pressure on the SCW, causing it to expand radially but to shrink longitudinally. This is thought to be achieved by the axial orientation of the cellulose microfibrils and the ability of matrix polymers to swell (Burgert and Fratzl, 2009). Alternatively, the longitudinal shrinkage hypothesis proposes that crystallization of microfibrils in the G-layer, among which matrix polysaccharides such as xyloglucan are trapped, causes strain on the cellulose lattice, generating tension. This tension spreads throughout the fibre due to contact of the G-layers with the SCW layers and it is maintained by xyloglucan cross-links acting as a ‘molecular muscle’ (Mellerowicz and Gorshkova, 2012; Mellerowicz *et al.*, 2008).

One of the most significant findings in this study was the distinct spatial and developmental deposition pattern of (1–4)-β-D-galactan in cell walls of G-fibres. (1–4)-β-D-galactan can be present as a side chain on RGI (Fagerstedt *et al.*, 2014) and it is considered to be a specific marker for both TW in angiosperms (Arend, 2008) and CW in gymnosperms (Altaner *et al.*, 2010). The latter have been shown to contain up to 10% of (1–4)-β-D-galactan. In softwoods, (1–4)-β-D-galactan has been detected with the LM5 antibody and found to be most intensely deposited during the development of the outer S2 layer of CW tracheids; however, the inner S2 layer next to the lumen remained unlabelled (Altaner *et al.*, 2010). In poplar, the LM5 antibody bound specifically to TW fibres of current and previous year wood and was concentrated in the junction between the G-layer and the SCW, the G-layer itself exhibiting only weak and diffuse labelling (Arend, 2008). It was suggested that (1–4)-β-D-galactan may have a cross-linking function between the G-layer and the SCW. By contrast, the labelling patterns in willow showed that the G-layer itself can be highly enriched in (1–4)-β-D-galactan, especially in G-fibres where the G-layer is still thickening. However, in some fibre files the junction between the G-layer and SCW was predominantly labelled, in agreement with the studies of poplar discussed above. The presence of (1–4)-β-D-galactan in fibres forming a G-layer is known to be stage specific (Gorshkova *et al.*, 2010; Mikshina *et al.*, 2012). Similarly, this study has observed that the deposition of (1–4)-β-D-galactan varies within the G-layer and with G-fibre maturation. In some plants (1–4)-β-D-galactan has not been significantly detected in TW. For example, G-fibre cell walls in sweetgum (*L. styraciflua*) were not significantly labelled with the LM5 antibody and light labelling was detected only in the primary cell wall/S1 junction and in the lumen, leading to the conclusion that RGI molecules, if present, do not possess galactan side chains (Bowling and Vaughn, 2008).

The gelatinous-type cell wall of flax (*Linum usitatissimum*) fibres is formed by the deposition of two sequential layers, the Gn-layer (inner new developing layer), which is remodelled into the outer mature G-layer (Gorshkova *et al.*, 2010). (1–4)-β-D-galactan associated with RGI is deposited into the developing Gn-layer as shown by LM5 antibody labelling. Its presence, in conjunction with the action of β-galactosidase enzymes, is implicated in determining the mechanical properties of phloem G-fibres in flax (Roach *et al.*, 2011). The model proposed by Roach *et al.* (2011) suggests that the abundant deposition of a high molecular weight galactan in the Gn-layer prevents cellulose microfibrils from becoming tightly associated, so they remain loosely packed during early G-layer development. During maturation of the G-layer this high molecular weight galactan is removed by a galactosidase enzyme (LuBGAL1), allowing a close association of cellulose microfibrils, which results in a highly crystalline matrix characteristic of mature G-layers. Some low molecular weight galactan still remains in the mature G-layer. The present immunolabelling analyses of willow TW showed that (1–4)-β-D-galactan was present in the actual G-layer of developing and maturing G-fibres, whereas in the inner very mature G-fibres (1–4)-β-D-galactan was mainly confined to the interface between the SCW and the G-layer, suggesting similarities in the G-layer maturation process to that described above for flax fibres and consistent with the proposed role in allowing deposition of cellulose microfibrils.

The present observations with the CCRC-M38 antibody indicated that de-esterified homogalacturonan is present mainly in cell corners between all cells, including fibre cells and in the compound middle lamella in TW, OW, and NW. Being a pectic polysaccharide, its main function is in cell adhesion, and this explains why it has been detected in immunocytochemical studies in other plants at the cell junctions and middle lamella (Bowling and Vaughn, 2008; Guillemin *et al.*, 2005; His *et al.*, 2001). In the present study, there was a clear difference in the immunolabelling pattern between the outer younger xylem and the inner mature xylem of the willow stem. In mature inner G-fibres, in addition to its presence in the cell junctions and middle lamella, homogalacturonan was also present in a narrow layer adjacent to the lumen, as indicated by immunofluorescence of thick unembedded sections and also ultrathin TEM sections. These observations are in agreement with those reported by Bowling and Vaughn (2008) in G-fibres of sweetgum (*L. styraciflua*), where the middle lamella/primary cell wall as well as the so-called ‘terminal lamella’ on the lumen side of the G-layer were strongly labelled, but the G-layer itself was not labelled.

The LM10 antibody, which recognizes unsubstituted and relatively low-substituted xylans (McCartney *et al.*, 2005), labelled only the SCWs of fibres, ray parenchyma, and xylem vessels of TW, OW, and NW in willow stems. It is well documented that xylan is the main hemicellulose present in SCWs and that it is absent from the G-layer itself (Bowling and Vaughn, 2008; Gorshkova *et al.*, 2010; Kaneda *et al.*, 2010). Work on *Populus tremula* L. using the LM10 antibody has demonstrated that xylan is present only in the SCWs and not in the G-layer (Kim and Daniel, 2012).

The present study found that mannan was strongly associated with TW and was present in SCWs of developing G-fibres and also in the G-layer of mature G-fibres. Mannan epitopes probed with an anti-β-(1–4)-D-mannan antibody have been detected in cell walls of hybrid poplar, mainly in the S2 layer of NW fibre cells (Kaneda *et al.*, 2010). Similarly, also in poplar, mannan epitopes were localized with the LM21 antibody mainly in the S2 layer of cell walls of fibres of OW and NW. Whereas the G-fibres of TW were very weakly labelled, some labelling was observed in some G-fibres and also in the G-layer itself in more mature fibres (Kim and Daniel, 2012). A transcriptome and metabolome study of poplar (Andersson-Gunneras *et al.*, 2006) showed that GDP-mannose-pyrophosphorylase, the enzyme required for the synthesis of GDP-mannose, was down-regulated in TW. Furthermore, the amount of mannan synthase mRNA was greatly reduced in TW in comparison to NW. This does not agree with the immunofluorescence observations of the present study, where fibre cell walls of OW and NW were very weakly labelled and there was a progression of mannan deposition in TW fibres. Mannan was strongly localized in the SCW initially, in young developing G-fibres, and later in the G-layer of fully mature G-fibres, but at weaker intensity,

*FLA12* and *COBL4* transcripts were highly up-regulated in TW in poplar (Andersson-Gunneras *et al.*, 2006). Here, probes designed to willow orthologues of *FLA12* and *COBL4* transcripts showed complete specificity in spatial distribution, being confined to developing G-fibre cells in TW regions. As is often the case in *in situ* hybridization experiments on large gene families, the possibility cannot be excluded that the probes designed to *SxFLA12* may have cross-hybridized to a lesser degree with very similar transcripts from the same gene family, despite the probe being designed to span mostly 3′UTR sequence. For *SxCOBL4*, a member of a much smaller gene family in comparison to *SxFLA12*, the results should be more unequivocal. For both gene targets, the authors’ unpublished RNA-seq results do, however, strongly support the up-regulation of these specific genes in TW. Both COBRA and FLA family proteins are known to be anchored in the plasma membrane and physically associated with cellulose microfibrils during their deposition (Liu *et al.*, 2015). It seems likely that the *FLA12* and *COBL4* proteins have specific properties that are required for deposition of the highly anisotropic cellulose microfibrils required to form G-layers, and this is consistent with the complete specificity of expression observed here. A study of the COBRA-like gene *COCOBL1*, which is highly expressed in the cambium region of the conifer *Cunninghamia lanceolata* (Chinese fir), revealed that this gene family is involved in the control of tissue architecture. The protein *CCOCBL1* was localized in the plasma membrane and cell wall, and when overexpressed in tobacco it altered the shape of the leaf by inducing anisotropic growth, resulting in a differential dorsoventral expansion of the leaf (Gao *et al.*, 2013).

**Fig. 9.**
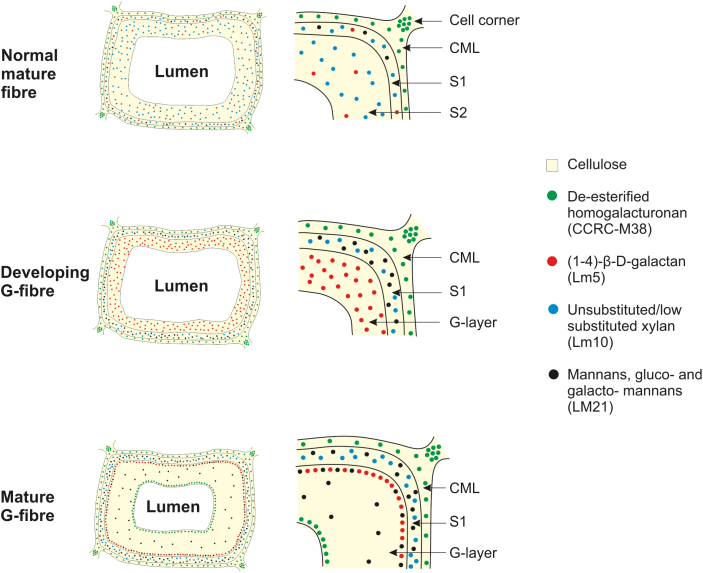
Generalized model of the distribution of β-galactan, xylan, homogalacturonan, and mannan epitopes in mature NW fibres (including OW fibres) and in developing and mature G-fibres in TW. Whole cells are shown on the left and detail of the cell wall is shown on the right. The pale yellow colour in the background in the cell wall represents cellulose, which is present in the cell wall layers in NW and TW fibres. The dots in four different colours represent the four cell wall polymers investigated in this study. The density of the dots gives an approximate indication of the strength of the labelling as seen by immunofluorescence and/or immunogold TEM. The homogalacturonan epitope (green dots) is present in NW and TW fibres mainly in the cell corners and also in the compound middle lamella (middle lamella + primary cell wall). Xylan (blue dots) is restricted to the SCWs of both NW and TW fibres, and it is completely absent from the G-layer in both developing and mature fibres. Red dots show the position of (1–4)-β-D-galactan, which is almost absent in the SCW of NW fibres, but is highly concentrated in the G-layer of developing fibres. In mature fibres, the red dots indicate that in most G-fibres, (1–4)-β-D-galactan is located predominantly in the interface between the SCW and the G-layer. The black dots indicate the location of mannans, which appear to be deposited in very small amounts in the SCW of NW fibres, but more intensely in developing G-fibres. Some mannan is also present in the G-layer of mature G-fibres.

The immunofluorescence and TEM observations of the present study can be used to derive a generalized model of the distribution of the (1–4)-β-D-galactan, homogalacturonan, xylan, and mannan epitopes (Fig. 9) in developing and mature G-fibres of TW of willow compared with NW. Although based on the detailed results obtained for one genotype of willow, in which TW, OW, and NW were compared, the authors have also studied one other genotype (data not shown) and found no clear differences in the results of immunolabelling and *in situ* hybridization. This suggests that the model will provide a useful generic representation to compare TW development in detail in willow genotypes differing in sugar release potential under RW-inducing conditions.

## Conclusions

Analyses of cell wall epitopes during the formation of TW in willow have allowed the development of a model of polysaccharide distribution in developing G-fibre cells. This shows (1–4)-β-D-galactan to be most abundant during G-layer development, which is consistent with a role in the deposition of cellulose microfibrils. This agrees with *in situ* hybridization, which shows specific labelling of G-fibre cells for *SxCOBL4* and *SxFLA12* transcripts, both of which are involved in cellulose microfibril deposition. These findings therefore suggest that all the features that are specific to G-fibre cells may contribute to the machinery that allows deposition of the highly anisotropic cellulose microfibrils in the G-layer, which is the defining characteristic of TW.

## Supplementary data

Supplementary data are available at *JXB* online.

Supplementary Table 1. Primers of *Sx*FLA12 and *SxCOBL4* for RNA probe synthesis in situ.

Supplementary Figure S1: Nucleotide sequence alignment of willow *SxFLA12* (A) and *SxCOBL4* (B) with their poplar orthologues.

Supplementary Figure S2. Negative immunolabelling controls of a 4-week inclined stem.

Supplementary Figure S3. Transverse sections of stems showing results of in situ hybridization experiments using SxFLA12 and control sense probes.

Supplementary Data

## Abbreviations:

CW: compression wood
DEPC: diethylpyrocarbonate
DIG: digoxigenin
FLA: fasciclin-like arabinogalactan
NBT/BCIP: nitro blue tetrazolium chloride/5-bromo-4-chloro-3-indolyl phosphate, toluidine salt
NW: normal wood
OW: opposite wood
RGI: rhamnogalacturonan I
RW: reaction wood
SCW: secondary cell wall
TEM: transmission electron microscopy
TW: tension wood.
