# Supplementary material for: G-fibre cell wall development in willow stems during tension wood induction
Source: J Exp Bot. 2015 Jul 28;66(20):6447–59. doi: 10.1093/jxb/erv358 (PMC4588891; doi:10.1093/jxb/erv358)
Supplement: Supplementary Data [file supp_66_20_6447__index.html]

G-fibre cell wall development in willow stems during tension wood induction — G-fibre cell wall development in willow stems during tension wood induction — Supplementary Data 

# G-fibre cell wall development in willow stems during tension wood induction

## Supplementary Data

Data files

- Supplementary Data - Supplementary Data
