# Supplementary material for: G-fibre cell wall development in willow stems during tension wood induction
Source: J Exp Bot. 2015 Jul 28;66(20):6447–59. doi: 10.1093/jxb/erv358 (PMC4588891; doi:10.1093/jxb/erv358)

# G-fibre cell wall development in willow stems during tension wood induction

Cristina Gritsch, Yongfang Wan, Rowan A.C. Mitchell, Peter R. Shewry, Steven J. Hanley, Angela Karp

**Supplementary figures and tables**

**Supplementary Figure S1:** Nucleotide sequence alignment of willow *SxFLA12* (A) and *SxCOBL4* (B) with their poplar orthologues.

**Supplementary Figure S2.** Negative immunolabeling controls of a 4-week inclined stem. No signal is present when the primary antibodies are omitted and sections are treated with the 488 and 633 Alexa secondary antibodies. No lignin autofluorescence is observed. TMP channel shows the transmission image of the section. Settings were the same as for experimental sections.

**Supplementary Figure S3.** Transverse sections of stems showing results of *in situ* hybridization experiments using *SxFLA12* and control sense probes. (A-B) Expression *SxFLA12* in the G-fibres in 1 and 4 week tipped stems (purple colour). (C-D) Opposite wood of the same stems as in A and B, showing no expression with the *SxFLA12* antisense probe. (E-F) No or very little expression of *SxFLA12* in normal wood (upright control stems). (G) 1-week tipped stem showing no signal in the TW side with the *SxCOBL4* sense probe. (H) 4-week tipped stem showing no signal in G-fibres. Bars: 100 µm.

**Supplementary Table 1**. Primers of *Sx*FLA12 and *SxCOBL4* for RNA probe synthesis in situ (underlined sequence was T3 or T7). SP: sense probe, AS: anti-sense probe.

| **Primers for genes** | **Sequences** |
|---|---|
| *SxCOBL4*-SP-T3 | AATTAACCCTCACTAAAGGGAGACAAGTTTCTTACTCCTGATGGTCTG |
| *SxCOBL4*-SP-R | ATTGAAGAAGTGAGCTGTTGTCTTT |
| *SxCOBL4*-AS-F | CAAGTTTCTTACTCCTGATGGTCTG |
| *SxCOBL4*-AS-T7 | GCTTCTAATACGACTCACTATAGGGAGAATTGAAGAAGTGAGCTGTTGTCTTT |
| *SxFLA12*-SP-T7 | GAATTGTAATACGACTCACTATAGGGTCCCAGTTTCAGACCGTAACTAA |
| *SxFLA12*-SP-R | CAATCAAGCCACCATTATCTAGC |
| *SxFLA12*-AS-F | TCCCAGTTTCAGACCGTAACTAA |
| *SxFLA12*-AS-T7 | GAATTGTAATACGACTCACTATAGGGCAATCAAGCCACCATTATCTAGC |

# Supplementary Figure S1A

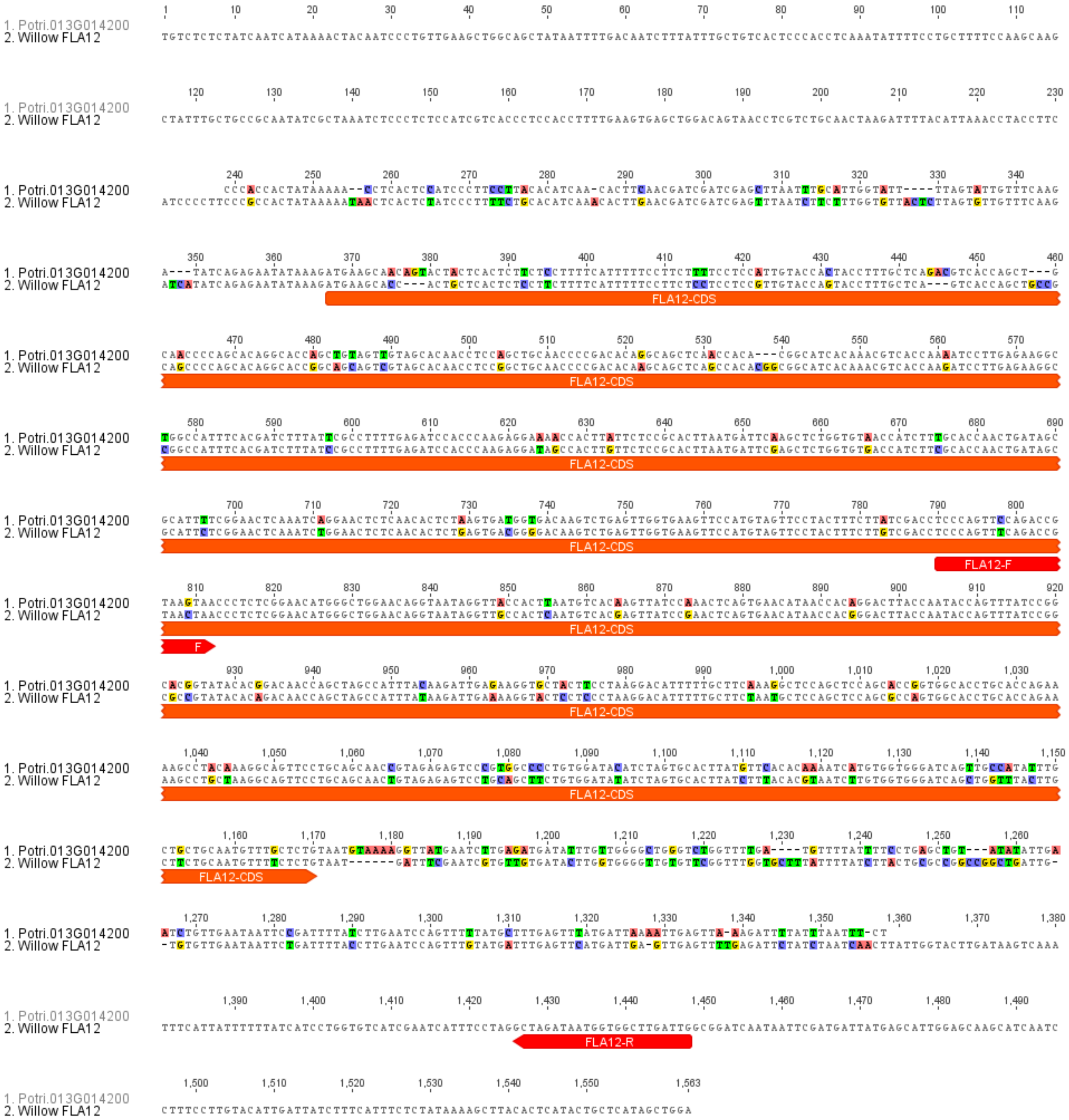

# Supplementary Figure S1B

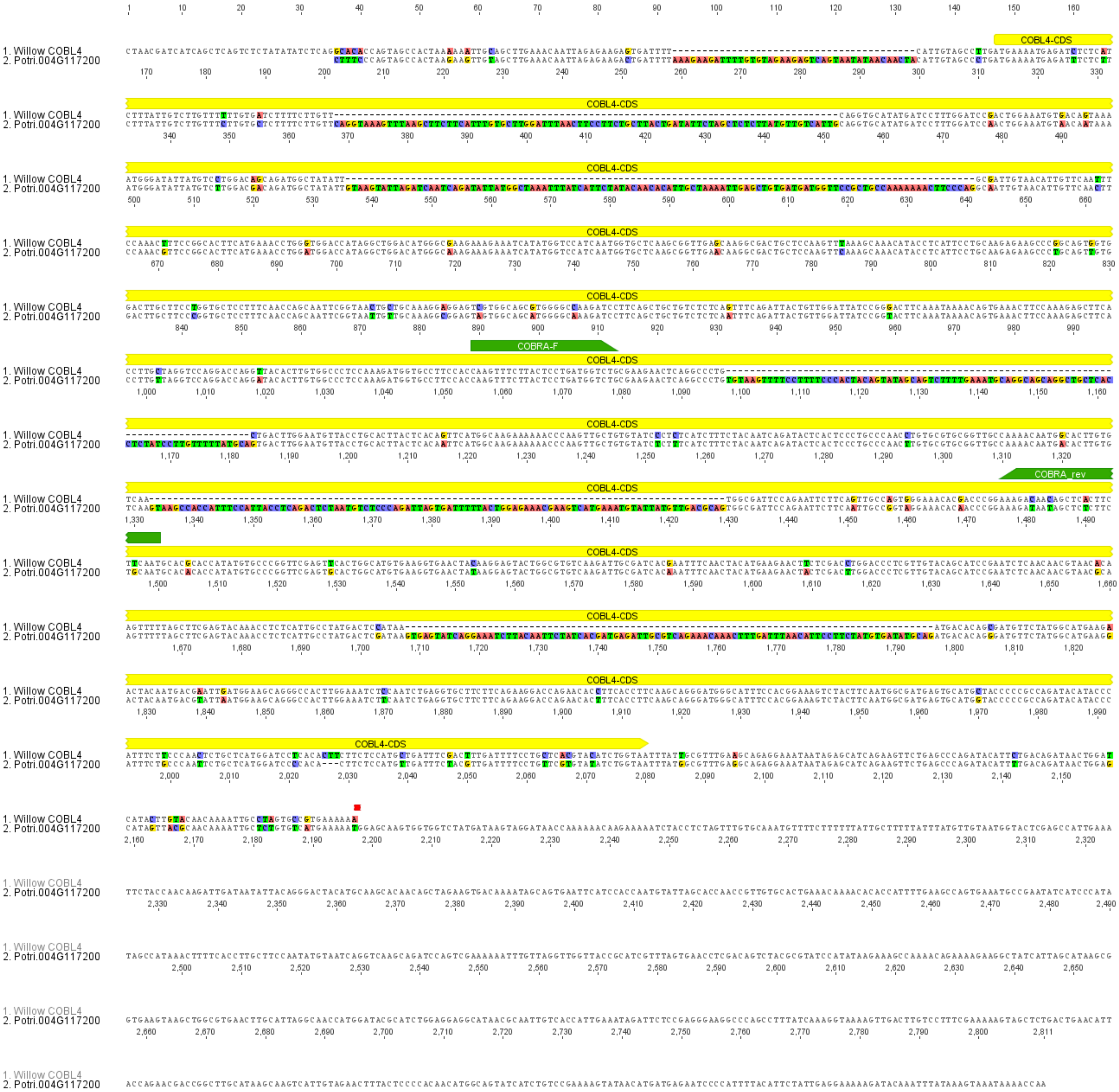

**Supplementary Fig S2 - Negative immunolabelling control - 4-week tipped Tension wood side resin section probed with secondary antibodies (Alexa 488 and Alexa 633) only**

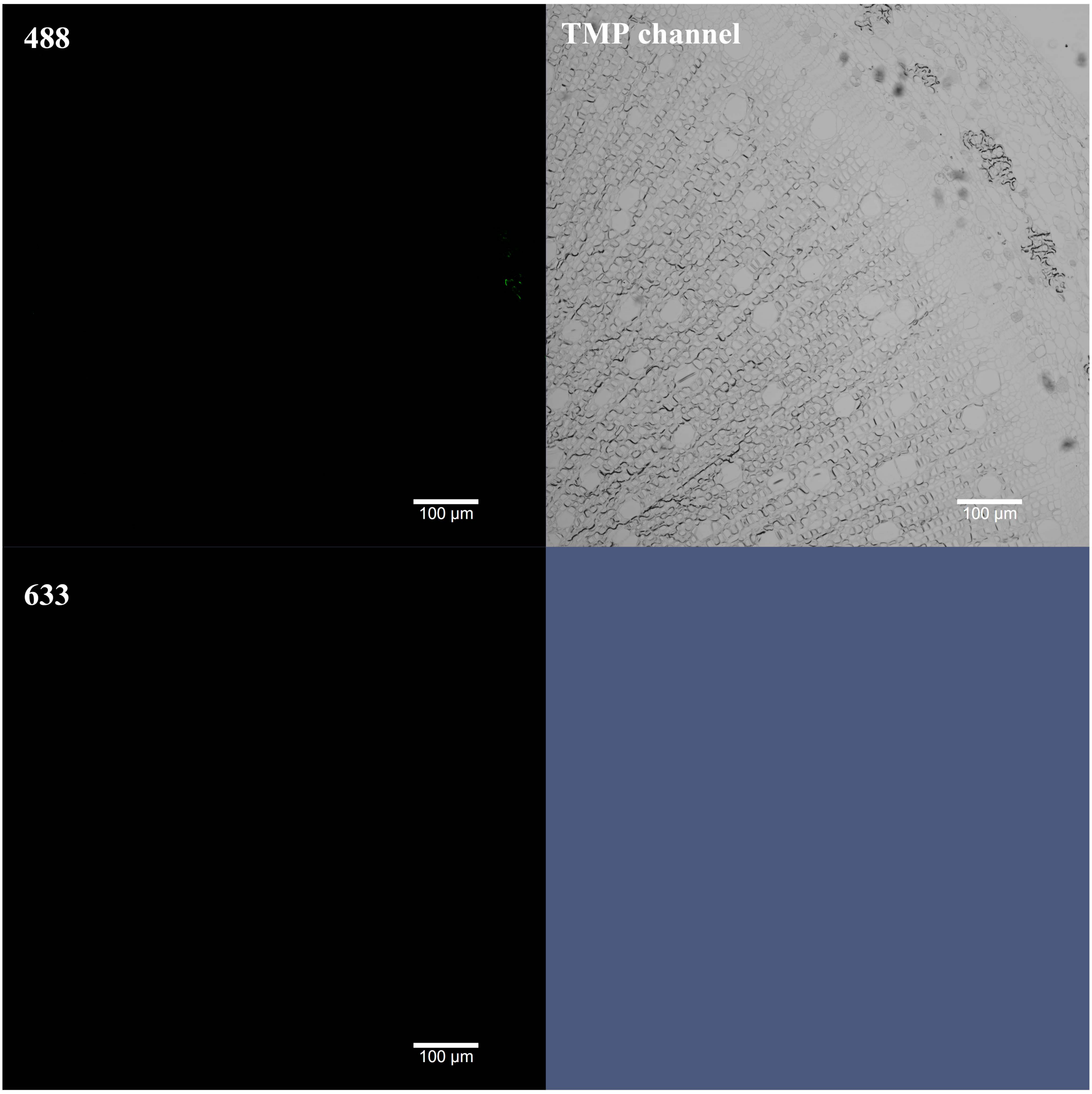

# Supplementary Fig S3 - in situ

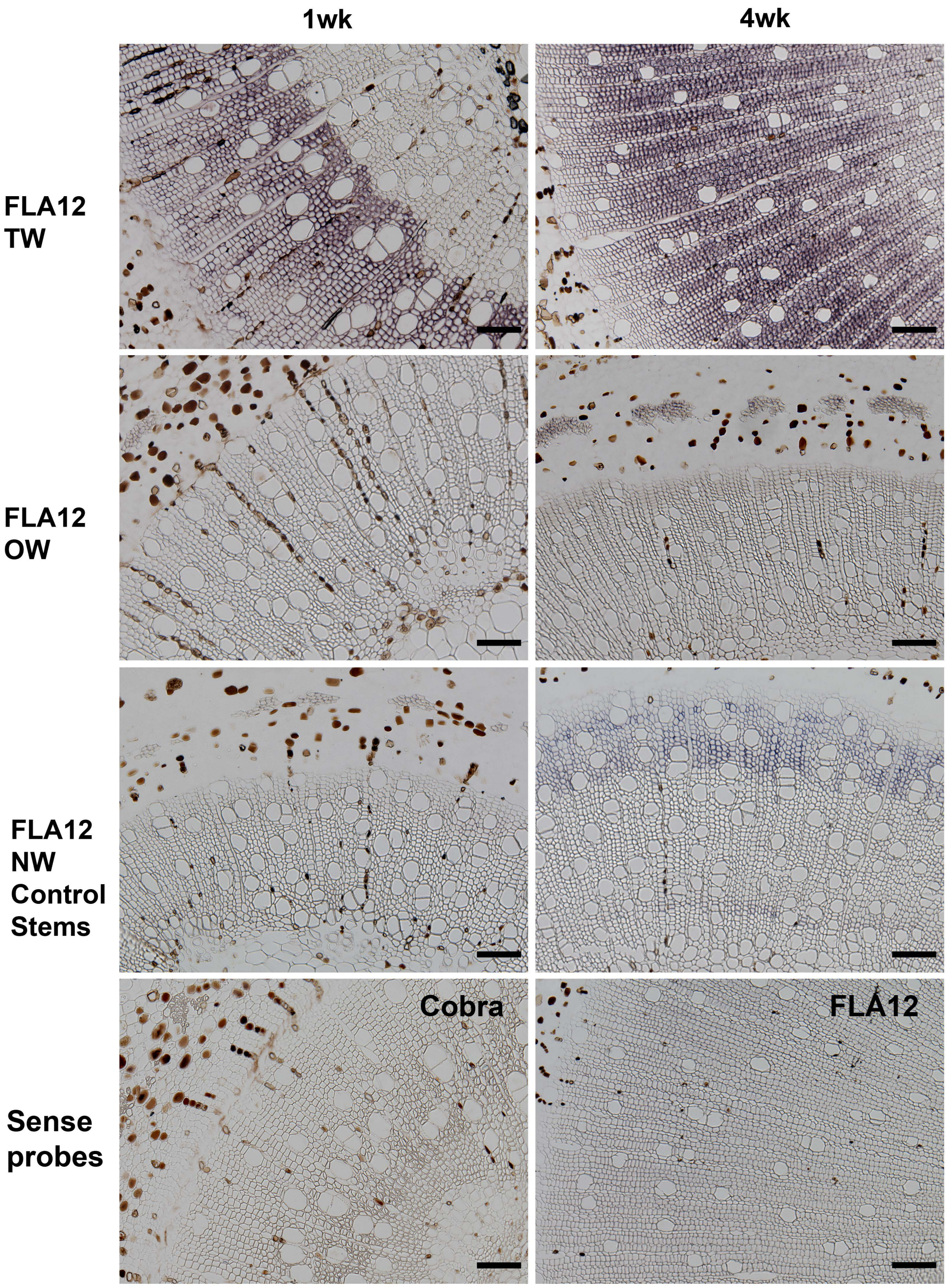

Supplement: Supplementary Data [file supp_erv358_jexbot152611_file001.pdf]
